# Risk of falls, vestibular multimodal processing, and multisensory integration decline in the elderly–Predictive role of the functional head impulse test

**DOI:** 10.3389/fneur.2022.964017

**Published:** 2022-11-16

**Authors:** Leandro Politi, Lorenzo Salerni, Luciano Bubbico, Fabio Ferretti, Mario Carucci, Giovanni Rubegni, Marco Mandalà

**Affiliations:** ^1^ENT Unit, San Donato Hospital, Arezzo, Italy; ^2^ENT Unit, Department of Medicine Surgery and Neuroscience, University of Siena, Siena, Italy; ^3^Department of Sensory Neural Disability Research, National Institute for Public Politic Analysis (INAPP), Rome, Italy

**Keywords:** elderly, falls, presbyastasis, cognitive decline, functional head impulse test

## Abstract

Age-related degeneration of the vestibular system, also known as presbyastasis, leads to unstable gait and higher risk of falls. These conditions affect lifestyle and may have non-negligible social repercussions due to fear-related states of anxiety and depression. In order to develop a model for predicting risk of falls, we assessed vestibulo-ocular function by video and functional Head Impulse Tests (vHIT and fHIT) and their possible correlations with Tinetti Balance Test score. Thirty-one patients over 65 years of age admitted with trauma due to falls were recruited. Vestibular evaluation (complete otoneurological assessment, vHIT, fHIT), cognitive tests (Mini Mental State Examination), anxiety and depression evaluation and Tinetti Balance Test were performed. The possibility of a correlation between the head impulse tests (vHIT, fHIT) and the Tinetti Balance Test was investigated by logistic regression analysis (Nagelkerke *r*^2^ and Wald test). A linear correlation was found between the Tinetti Balance Test score and fHIT, whereas no correlation was found for vHIT. Functional HIT is an effective test for predicting the risk of falls in elderly patients.

## Introduction

Age-related degeneration of the vestibular system is a condition leading to unstable gait, dizziness and an increased risk of falls in the elderly, reducing everyday activities and interfering with lifestyle. Dizziness and gait abnormalities in the elderly are consequences of a correlation between progressive multifactorial conditions due to vestibular sensorial deficits (paroxysmal positional vertigo, unilateral or bilateral vestibular loss, Meniere's disease), central disorders (neurodegenerative or vascular disorders, cerebellar hypofunction, ataxia), anxiety, phobic vertigo and cognitive loss, all events that may be enhanced by comorbidities (cardiovascular, visual, musculoskeletal and pharmacological). The term presbyastasis summarizes these conditions and can be defined as age-related imbalance of the peripheral and central balance systems (vestibular, visual, somatosensory) involved in maintaining correct balance.

Disequilibrium is a fairly common problem in the elderly. In numerous studies (1–3) it has been shown that one third of persons over 60 years of age and one half over 85 years have disorders requiring medical observation. According to WHO, 30% of people over 65, and 50% of those over 80, fall at least once a year (3). Disturbances of equilibrium also entail an increase in the risk of falls and fractures in the elderly, limiting physical activity with non-negligible social repercussions (4) and fear-related states of anxiety or depression.

Clinical assessment and testing of equilibrium in the elderly should therefore consider multiple tasks, including vestibulo-ocular and motor-cognitive. An ideal test to assess the risk of falling or gait abnormalities must be simple, short, repeatable to allow follow-up and readily extended to a large population. The Tinetti Balance Test can be considered sensitive for assessing balance, gait and fear of falling (5, 6), although its specificity is low. Evaluation of the vestibulo-ocular reflex (VOR) is essential for studying patients with disequilibrium or dizziness. Unilateral and especially bilateral vestibular loss with severe unsteadiness and oscillopsia can be considered “limit” conditions in which compensation capacity is reduced and can be easily detected by bedside examination (head impulse test, head heave test, skew), video head impulse test (vHIT) or caloric irrigation. In the elderly, VOR usually undergoes slow steady asymmetrical decline, not easily detected when silent.

Recently, the functional head impulse test (fHIT) was introduced among other laboratory vestibular tests (7–10). Since fHIT is a subjective functional test and does not require analysis of eye movements as does vHIT, it also investigates the performance of the vestibular ocular reflex at a higher level in the brain (vestibular/associative cortex). In particular, fHIT explores the ability of the subject to correctly identify an image presented briefly during VOR activation. This characteristic of fHIT, associated with the simplicity and speed of performing it and analyzing its results, could be important for developing a new screening test for subjects at risk of falls. Functional HIT may have a key role in identifying subjects suffering from vestibular multimodal processing and multi-sensory integration decline that may lead to an increased risk of falls (11).

The main purpose of our study is to assess the risk of falling in a group of patients with recurrent falls and in age matched controls using the outcomes of vestibular tests (vHIT and fHIT).

The secondary objectives are: (a) analyze the correlation between fHIT and Tinetti's scores with some individual characteristics (age, cognitive impairment measured by Mini Mental State Examination, and the impact of dizziness on daily life assessed with Dizziness Handicap Index); (b) compare the relationships between Tinetti/DHI scores and fHIT/vHIT scores in cases and controls; (c) the power of fHIT and Tinetti scores in predicting falls.

## Materials and methods

We conducted a case control study on patients over 65 years of age who presented for emergency care at Siena University Hospital with trauma due to accidental falls in the period January 2019 to December 2019. Exclusion criteria were: documented vestibular pathologies [positional vertigo, vestibular neuritis, Menière disease (12)], anopsia/hemianopsia, central nervous system and inner ear disorders. The control group consisted of patients over 65 years of age evaluated at the ENT department for conditions not involving vertigo, dizziness, unstable gait or falls. The control group met the same exclusion criteria as the case group and had not suffered any falls in the last year. The study was approved by the local ethics committee and all procedures complied with the Declaration of Helsinki. Patients signed informed consent before participating in the study.

Patients in the case and control group were evaluated on the day of admission to the emergency care unit or ENT Department. Patients and controls underwent:

- Bedside otoneurological examination;
- Detailed medical history, focusing on: number of injurious falls (falls associated with trauma such as haematomas, dislocations or fractures) last year/in the last 5 years, episodes of vertigo/instability and their duration, use of medications, visual disturbances, urinary dysfunction, previous brain MRIs, occupation, education and social conditions such as poverty, solitude, widowhood;
- Tinetti Balance Test (13);
- Dizziness Handicap Inventory (DHI) (14);
- Hospital Anxiety and Depression Scale (HADS) (15);
- Mini-Mental State Examination, adjusted for age and education (16);
- Evaluation of VOR by vHIT (Otometrics ICS Impulse USB and OTOsuite Vestibular Software analysis);
- Evaluation of VOR by fHIT (fHIT 1.0 ver. B01, Beon Solutions srl).

**Figure 1:**
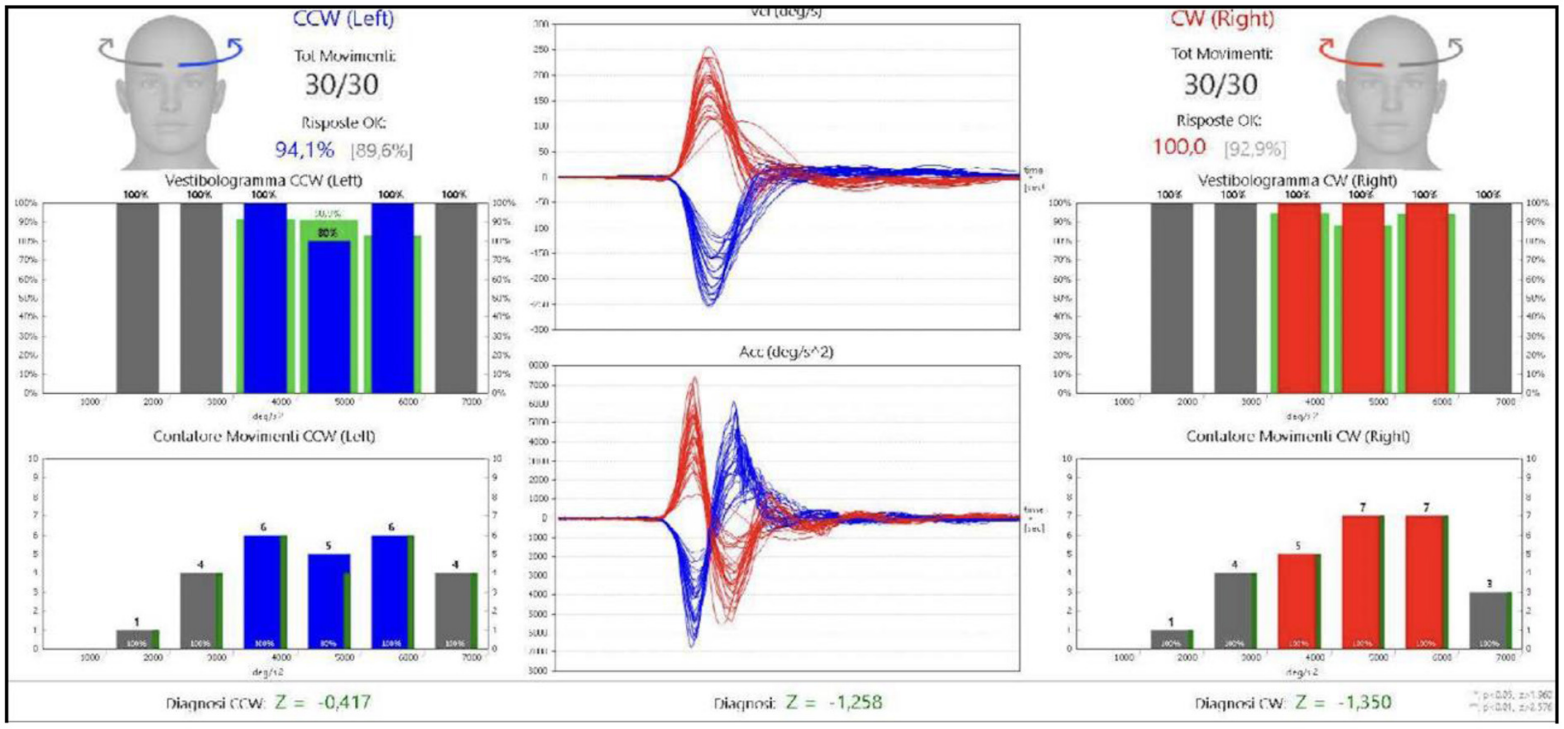
fHIT results in normal patient.

**Figure 2:**
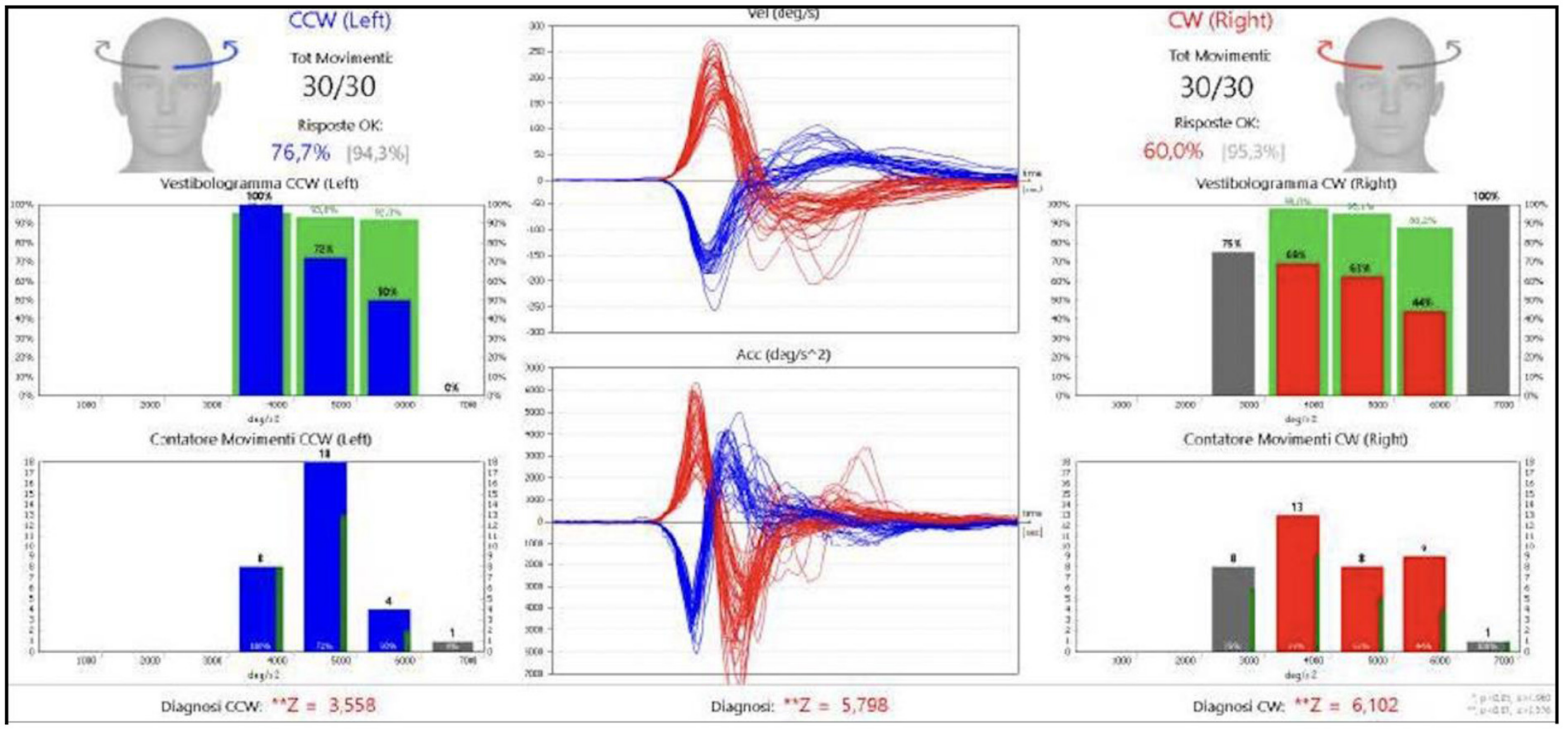
fHIT results in pathological patient.

An acquaintance or relative was always present to help patients during recording of medical history. To perform fHIT, the examiner turned the patient's head abruptly and unpredictably in the plane of a pair of semicircular canals, about 15° in about 100 ms. Each trial was recorded and classified in terms of peak acceleration by the accelerometer placed on the subject's forehead. During peak acceleration, an optotype (Landolt's C) briefly (80 ms) appeared on a high-refresh-rate monitor and the subject had to identify the correct orientation of the optotype and press the correct key on a pad. Optotype size was normalized and adapted to individual visual acuity according to the manufacturer's instructions. We conducted 30 trials per side. The number of correct answers was expressed as a percentage. Functional HIT provides information on the patient's ability to stabilize images on the fovea during unpredictable high frequency vestibular stimuli and central multimodal processing of multi-sensory input (Figures 1, 2).

The video head impulse test (vHIT) is the computerized version of the clinical head impulse test. A high-performance head-mounted infrared camera records eye movements, while the velocity/acceleration of imposed head thrust is recorded by an accelerometer. It is currently the elective procedure for determining VOR performance.

We compared vHIT and fHIT results within and between groups. We considered VOR gain <0.80 by vHIT from the better ear and responses to fHIT ≤ 80% on the better side to be pathological, as specified by the manufacturer (17). For simplicity and compliance, we only tested VOR of the lateral semicircular canal.

The Tinetti Balance Test was performed on all patients. The score has a “balance section” and a “gait section” with ten and seven items, respectively. To evaluate balance the patient sat on a hard, armless, stable chair. The patient was then asked to rise from seated position without using the arms or hands. Next, while the patient was standing, the examiner asked the patient to move his/her feet as close together as possible. The examiner then pressed three times on the patient's sternum with the palm of the hand. This was done once with the patient's eyes open and then with the eyes closed. Lastly, the patient was asked to make a 360 degree turn and sit back down on the chair. The scoring system for this portion was broken down into ten standardized scoring subsets to make a total score of sixteen.

To assesses the patient's gait the patient was told to walk about 3 m at a regular pace and then turn around to walk back to the starting point at a quick but safe pace. The scoring system for this portion was broken down into seven standardized scoring subsets to give a total score of twelve.

The scores from both portions of the examination were added together to obtain a total score (maximum total score = 28), <18 indicating high, 19–23 moderate and ≥24 low risk of falling (18). Those with a score <24 were classified at risk of falling, as indicated by the guidelines “Prevention of falls from domestic accidents in the elderly” (6).

**Figure 3:**
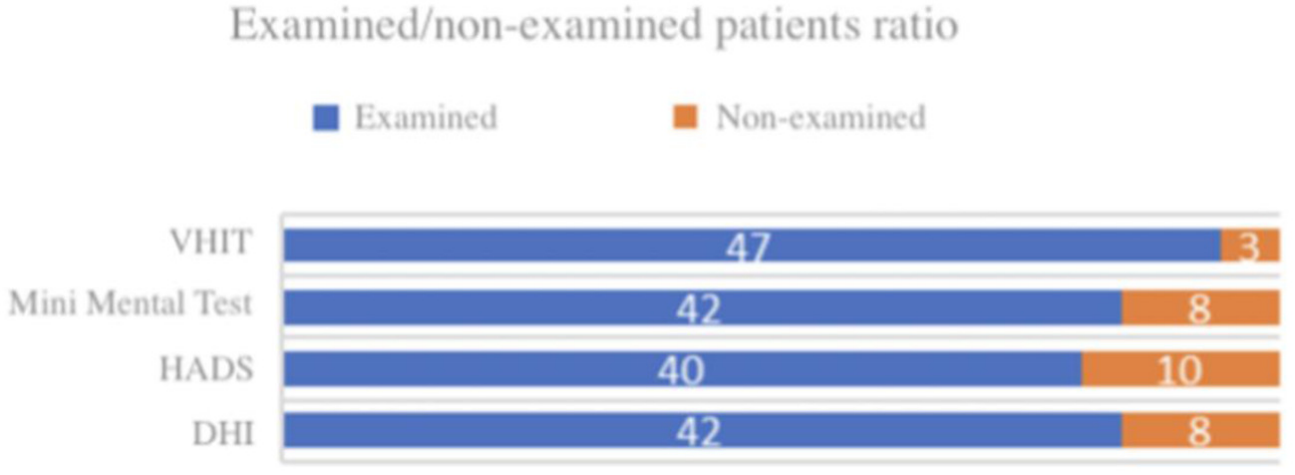
Subjects examined/not evaluated by vHIT, MMT, HADS, and DHI.

The Tinetti Balance Test scores and fHIT responses of all subjects were evaluated. In some subjects it was not possible to perform vHIT, DHI, HADS and/or the Mini-Mental test for questions of time and patient fatigue (Figure 3).

### Statistical analysis

The data was summarized with descriptive statistics (mean and standard deviations for continuous measures, frequency and relative frequency for categorical data). Individual characteristics of cases and controls were compared using parametric and non-parametric tests, according to the type of measure and whether the continuous variables showed a normal distribution (checked by Student's *t* test for independent samples, Mann-Whitney U test and Fisher exact test). The odds ratio was assessed, considering the number of falls in the previous year or the last 5 years as exposure, and a fHIT response ≤ 0.80 as outcome. The 95% confidence interval was calculated and a significance test was conducted to verify the null hypothesis of odds ratio equal to 1 (Z test). Since most of the variables showed non-normal distribution, non-parametric correlations were used to examine the relationship between Tinetti score and fHIT response vs. age, and between Mini-Mental State and falls in the last year or 5 years. The same method was used to analyse the relationship between Tinetti and HDI vs. fHIT and vHIT responses separately in cases and controls. Lastly, the predictive power of Tinetti and fHIT with respect to the occurrence of falls was assessed by binary logistic regression with a dichotomous dependent variable (“falls in the last year” or “falls in the last 5 years” - YES/NO), estimating a series of models with predictors represented by the Tinetti score or the fHIT response. Due to the high correlation between Tinetti and fHIT, the estimation of a multivariate model with both variables as predictors was not attempted. Goodness of fit was assessed by the Hosmer-Lemeshow test and by Nagelkerke's *R*^2^. Wald statistics were considered to determine the relevance of each independent variable in predicting the event “falls.”

Data analysis was performed with IBM-SPSS Statistics Version 25.0 (Release 2017; IBM Corp., Armonk, New York). Significance was set at *p* < 0.05.

## Results

Our study population consisted of 31 cases and 19 controls. The features of the groups are compared in Table 1. Gender did not differ significantly between the two groups: 45.2% of cases and 36.8% of controls were female (Fisher exact test, *p* = 0.768). The same was true for age: cases 79.16 ± 1.32 years, controls 78.00 ± 2.21 years (*p* = 0.222), DHI: cases 32.80 ± 5.26, controls 19.86 ± 4.13 (*p* = 0.318), HADS-Anxiety: cases 8.64 ± 0.89, controls 6.64 ± 1.09 (*p* = 0.145), HADS-Depression: cases 8.56 ± 0.95, controls 6.93 ± 0.98 (*p* = 0.229) and Mini-Mental State Examination: cases 27.31 ± 0.34, controls 26.94 ± 0.86 (*p* = 0.547). The risk of falls was significantly higher in the case group: the Tinetti score was 18.80 ± 1.23 in the case group and 23.00 ± 1.75 in controls (*p* = 0.003), while fHIT was 66.40% ± 5.12 and 76.79% ± 7.04, respectively (*p* = 0.029). The measures of vHIT were not significantly different in the two groups: cases 0.97 ± 0.04, controls 0.87 ± 0.05 (*p* = 0.131). The mean number of falls reported in the last year was 0.24 ± 0.25 and in the last 5 years 1.68 ± 0.18. Because of the study design, the number of falls in the control group was zero since they were not considered to be exposed to falls.

**Table 1:** Age, Tinetti Balance Test, fHIT, DHI, HADS, Mini-Mental Test scores, and falls during last year/last 5 years; data are expressed in mean and (standard deviation) for continuous measure or frequency and relative frequencies for categorical measures.

	**Case**	**Control**	**Statistic**	***P*-value**
Age	79.16 (1.32)	78.00 (2.21)	M-W U = 233.500	0.222*
**Gender**
F	14 (45.16%)	7 (36.84%)		0.768**
M	17 (54.84%)	12 (63.16%)		
Tinetti	18.80 (1.23)	23.00 (1.75)	M-W U = 145.000	0.003*
fHIT	66.40% (5.12%)	76.79% (7.04%)	M-W U = 186.000	0.029*
vHIT	0.97 (0.04)	0.87 (0.05)	*t*_(45)_ = 1.537	0.131***
DHI	32.80 (5.26)	19.86 (4.13)	M-W U = 164.500	0.318*
HADS (Anxiety)	8.64 (0.89)	6.64 (1.09)	*t*_(38)_ = 1.488	0.145***
HADS (Depression)	8.56 (0.95)	6.93 (0.98)	*t*_(38)_ = 1.222	0.229***
Mini-Mental State	27.31 (0.34)	26.94 (0.86)	M-W U = 173.500	0.547*
Falls-last year	1.24 (0.25)	0.00 (0.00)		
Falls-last 5 years	1.68 (0.18)	0.00 (0.00)		

*Mann-Whitney U test. **Fisher Exact test. ***t-Student test for independent samples.

The results of the odds ratio showed that those who reported falls in the last year had 3.9 times the odds (95% CI: 1.191–12.681) of having an fHIT <0.80. The corresponding odds ratio for falls in the last 5 years was 2.7 (95% CI: 0.841–8.311). The null hypothesis related to an odds ratio of 1 was rejected in both situations (last year and last 5 years), although weak evidence was found in the latter case (*p* = 0.012 and *p* = 0.048, respectively).

Table 2 shows correlations between Tinetti scores and fHIT percentages vs. age, Mini-Mental State scores, number of falls in the last year, and number of falls in the last 5 years. The risk of falls measured with Tinetti and fHIT was inversely and significantly correlated with age: the lower the score/percentage, the higher the age of the patient (Tinetti: ρ = −0.516, *p* = 0.000; fHIT: ρ = −0.560, *p* = 0.000). Cognitive assessment (Mini-Mental State score) did not show any significant correlation with the risk of falls measures, but a significant positive correlation was found between Tinetti score and falls in the last 5 years (ρ = −0.304, *p* = 0.032), and between fHIT and falls in the last year (ρ = −0.313, *p* = 0.027).

**Table 2:** Non-parametric correlations between Tinetti/fHit scores and age, Mini-Mental State score, falls occurred during the last year and falls occurred during the last 5 years.

	**Tinetti**		**fHIT**	
	**Spearman's ρ**	***P*-value**	**Spearman's ρ**	***P*-value**
Age	−0.516	**0.000**	−0.560	**0.000**
Mini-Mental State	0.082	0.607	−0.215	0.172
Falls-last year	−0.261	0.067	−0.313	**0.027**
Falls-last 5 years	−0.304	**0.032**	−0.168	0.245

Bold characters are the statistically significant values.

Table 3 summarizes the results of the non-parametric correlations of the Tinetti and DHI scores with fHIT and vHIT in cases and controls separately. DHI values were not correlated with fHIT or vHIT responses in either group. A significant positive correlation was found between Tinetti scores and fHIT in the case group and in the control group (ρ = 0.629, *p* = 0.000 and ρ = 0.734, *p* = 0.000, respectively). The correlation between Tinetti score and vHIT response was significant only in controls (ρ = 0.621, *p* = 0.008).

**Table 3:** Non-parametric correlations between Tinetti/DHI scores and fHIT/vHIT in cases and controls.

		**Tinetti**		**DHI**	
		**Spearman's rho**	***P*-value**	**Spearman's rho**	***P*-value**
fHIT	Cases	0.629	**0.000**	−0.165	0.412
	Controls	0.734	**0.000**	−0.369	0.176
vHIT	Cases	0.349	0.059	−0.159	0.439
	Controls	0.621	**0.008**	−0.024	0.939

Bold characters are the statistically significant values.

We conservatively set the normality threshold at fHIT >80% on the better side. To examine the predictive power of the risk of falls assessment tools (Tinetti and fHIT), we used binary logistic regressions with a dichotomous dependent variable (“falls in the last year” or “falls in the last 5 years” - YES/NO), estimating two multivariate models (with Tinetti and fHIT results as predictors).

Table 4 shows the results of the binary logistic regression with falls in the last year as dependent variable and Tinetti score as predictor. The model correctly classified 68.4% of subjects. Although fit was good (Hosmer-Lemeshow test not significant, *p* = 0.498), Nagelkerke's *R*^2^ was quite poor (0.104). The predictor parameter was not significant (Tinetti: β = −0.093, *p* = 0.093) and showed a Wald statistic of 2.828. The negative sign of the parameter highlighted that the probability of falling decreases as the Tinetti score increases.

**Table 4:** Results of the binary logistic regression with falls during the last year as the dependent variable (dichotomous: YES/NO); Tinetti score as predictor.

	**B**	**S.E**.	**Wald**	***P-*value**
Tinetti	−0.093	0.055	2.828	0.093
Constant	1.408	0.969	2.11	0.146

Table 5 describes the results of the same previous model, but with fHIT as predictor: 71.1% of subjects were correctly classified. Goodness of fit was confirmed by a non-significant Hosmer-Lemeshow result (*p* = 0.531) and Nagelkerke's *R*^2^ was higher than for the previous model (0.178) but still quite low. The predictor parameter was significant (β = −3.271, *p* = 0.033) and showed a Wald statistic of 4.569. Again the sign of the fHIT parameter was negative, indicating that the probability of falling increases with decreasing fHIT percentage.

**Table 5:** Results of the binary logistic regression with falls during the last year as the dependent variable (dichotomous: YES/NO); fHIT score as predictor.

	**B**	**S.E**.	**Wald**	***P*-value**
fHIT	−3.271	1.53	4.569	0.033
Constant	2.48	1.191	4.335	0.037

The two models shared an acceptable goodness of fit according to the Hosmer-Lemeshow test but showed little improvement in model likelihood over a null model (expressed by Nagelkerke's *R*^2^). These results were not contradictory: the first gave an overall model evaluation, while the second provided information about the validation of the model, and the second was more sensitive to sample size.

The same two models were fitted using falls during the last 5 years as dependent variable. Summarizing the results, fHIT was not significantly predictive, while the model with the Tinetti scale obtained significant results (b = −0.097, *p* = 0.046; Wald = 3.982; percentage of correctly predicted cases: 66%; Nagelkerke's *R*^2^ = 0.113).

## Discussion

Balance disorders, especially in the elderly, are a public health problem, not only due to direct costs, but also to indirect social and rehabilitation costs. Direct medical costs for fatal and non-fatal falls in the USA have been estimated at about $616.5 million and $30.3 billion, respectively, of which 57% ($17.2 billion) of the total was for hospitalizations (19).

Risk assessment in elderly patients is a reference point for preventive medicine. Based on the literature and the specific Italian context, we confirmed that the Tinetti Balance Test is one of the best tools for assessing balance in elderly patients. However, the best predictive cut-offs for this specific scale should be defined by identifying subjects at high risk of falls in larger and more representative samples of the elderly general population (6).

Older age is marked by degeneration of different neuronal structures, including those responsible for maintaining equilibrium, such as vestibular receptors, central vestibular neurons, the visual system, the proprioceptive system and the corresponding integrative structures. Histopathological studies have shown a direct correlation between progressive rarefaction of semicircular canal ciliated cells and age, while loss of saccule and utricle hair cells is less significant (20). Indeed, analysis of the vestibulo-ocular reflex (VOR), confirmed by video HIT, have shown a progressive reduction in VOR in elderly subjects, linked to gradual loss of the ability to compensate rapid rotations of the head with eye movements (21).

Though essential in the diagnosis of vestibular pathologies, vHIT does not appear to be the test of choice in the framework of presbyastasis. Elderly patients with normal vHIT results may have difficulty seeing clearly during head movements, due to problems with integration of vestibular and visual afferents. It is in this field that fHIT is most useful. Although fHIT and vHIT both investigate VOR, their results diverge in elderly patients at risk of falling. In fact, in our study, VOR gain assessed by vHIT did not differ significantly in cases and controls. In contrast, we found a statistically significant difference between cases and controls in term of outcomes with fHIT and the Tinetti score. Functional HIT showed a strong correlation with risk of falling in the last year: patients with fHIT below 80% had ~3.9 times the odds of a fall in the previous year than those with fHIT below 80%.

In our analysis, we went further, estimating the power of fHIT and Tinetti as risk of falls assessment tools. Surprisingly, we found that in our binary logistic regression models, only fHIT was significant as a predictor of risk of falls.

These results may be explained by the fact that correct optotype identification by fHIT not only requires an adequate VOR, but also an adequate set of cortical, vestibular and visual associative nerve structures. Functional HIT investigates the highest, most complex and multimodal processing of the vestibular-ocular reflex and its central multisensory integration which have been studied relatively little in presbyastasis (22). A pathological fHIT result, without VOR gain alterations, is an indication for rehabilitation in elderly subjects, because it is correlated with a high risk of falls.

Functional HIT proved to be a useful, easy-to-perform diagnostic test in the study of elderly patients at risk of falling. The fHIT data collected in our study showed an excellent correlation with Tinetti Balance Test score, both in patients with documented falls, and in patients at risk of falling. In fact, a deteriorating fHIT was associated with a worsening Tinetti score. We also found an inverse correlation between fHIT percentage and Tinetti score vs. age, indicating that Tinetti score and fHIT percentage decrease with increasing patient age. We highlight that fHIT and Tinetti test results were not correlated with the Mini-Mental-State score, which may indicate that vestibular decline is independent of global intellectual decline.

## Conclusion

Recent findings demonstrate that cognitive decline is a primary factor in determining the risk of falls. For older patients with subjective cognitive impairment and unstable gait, a motor-cognitive risk syndrome has been postulated (23). Walking while solving cognitive tasks becomes harder in the elderly, as do spatial memory, mental navigation and imagery (24), underlining the link between the vestibular system and higher cognitive processes (25, 26). A predictive tool for risk of falls in the elderly, such as fHIT, could estimate vestibular multimodal processing decline and therefore play an important role in prevention and in assessing rehabilitation targets. Future research is recommended to investigate correlations between fHIT results and other forms of cognitive impairment.

## Data availability statement

The original contributions presented in the study are included in the article/supplementary material, further inquiries can be directed to the corresponding author.

## Ethics statement

Ethical review and approval was not required for the study on human participants in accordance with the local legislation and institutional requirements. The patients/participants provided their written informed consent to participate in this study.

## Author contributions

MM, LS, GR, MC, LB, FF, and LP: data analysis and article writing. LP: data collection. All authors contributed to the article and approved the submitted version.

## Conflict of interest

The authors declare that the research was conducted in the absence of any commercial or financial relationships that could be construed as a potential conflict of interest.

## Publisher's note

All claims expressed in this article are solely those of the authors and do not necessarily represent those of their affiliated organizations, or those of the publisher, the editors and the reviewers. Any product that may be evaluated in this article, or claim that may be made by its manufacturer, is not guaranteed or endorsed by the publisher.
